# S-induced modifications of the optoelectronic properties of ZnO mesoporous nanobelts

**DOI:** 10.1038/srep27948

**Published:** 2016-06-15

**Authors:** Filippo Fabbri, Lucia Nasi, Paolo Fedeli, Patrizia Ferro, Giancarlo Salviati, Roberto Mosca, Arrigo Calzolari, Alessandra Catellani

**Affiliations:** 1 CNR-IMEM, Parco Area delle Scienze 37a, I-43124 Parma, Italy; 2 KET Lab, c/o Italian Space Agency via del Politecnico, 00133 Roma, Italy; 3 CNR-NANO, Istituto Nanoscienze, Centro S3, 41125 Modena, Italy

## Abstract

The synthesis of ZnO porous nanobelts with high surface-to-volume ratio is envisaged to enhance the zinc oxide sensing and photocatalytic properties. Yet, controlled stoichiometry, doping and compensation of as-grown n-type behavior remain open problems for this compound. Here, we demonstrate the effect of residual sulfur atoms on the optical properties of ZnO highly porous, albeit purely wurtzite, nanobelts synthesized by solvothermal decomposition of ZnS hybrids. By means of combined cathodoluminescence analyses and density functional theory calculations, we attribute a feature appearing at 2.36 eV in the optical emission spectra to sulfur related intra-gap states. A comparison of different sulfur configurations in the ZnO matrix demonstrates the complex compensating effect on the electronic properties of the system induced by S-inclusion.

Zinc oxide (ZnO) one-dimensional nanostructures are of great interest for the development of devices in different fields, as energy harvesting, optoelectronic devices like light emitting diodes and solid state lasers^1^,^2^. Among these applications, ZnO porous nanostructures demonstrated superior performances in photocatalysis, water-splitting and bio-/gas-sensing due to the higher surface-volume ratio^3^,^4^,^5^. Furthermore, different works reported that modification of the electronic properties in the gap region of porous ZnO nanostructures can improve the water splitting efficiency and the gas-sensing response^6^,^7^. The capability to control the optoelectronic properties is in general highly desirable: this is particularly true for ZnO, since the as-grown samples generally present *n*-type characteristics induced by stoichiometric defects and uninitentional doping^8^,^9^.

One way to modify the ZnO optoelectronic properties and reactivity is to act on the states of the gap region, through co-doping or any compensation effect that restores the charge balance taking the Fermi level back to mid-gap. Sulfur incorporation in ZnO is expected to modify the host optical properties^10^ and allow for energy gap (E_g_) engineering^10^,^11^,^12^ because of the larger E_g_ of ZnS (3.66 eV) compared to that of ZnO. Regardless the numerous advantages offered be eventual S doping, the related drawbacks have until now prohibited sulfur incorporation as useful dopant in ZnO: the differences in size and electronegativity between S and O can induce severe deformations in the host lattice; the problem is worsened by the significant difference between S evaporation and ZnO growth temperatures. Moreover, S can combine either with the constituent elements of the host (zinc and oxygen) or with impurities present in the sample (e.g. hydrogen), forming a number of different intermediate phases like SO_2_, S_2_O, H_2_S, H_2_SO_3_, ZnSO_4_, which lead to undesired structural defects. Beyond these technological problems in the synthesis, the effect of S-doping on the charge carrier character or the optical properties of ZnO nanostructures and the capability to efficiently induce *p*-doping has been debated for a long time: while including sulfur has been evoked as a possible route to enhance Nitrogen induced *p*-doping^10^, different works often describe S as responsible for *n*-doping of ZnO samples^13^,^14^.

In this work we clarify the effect of sulfur content on the optical properties in ZnO porous nanobelts (NBs) by comparing experimental results and first principles calculations. By using energy disperse X-ray spectroscopy and chemical mapping, we show that S is stable and homogeneously distributed in the ZnO matrix. Sulfur doping induces an intra-gap state that is responsible for an optical emission at 2.36 eV, as obtained by cathodoluminescence spectroscopy. Density functional theory (DFT) simulations confirm that S inclusions (both in substitutional O sites and as interstitial defects in the ZnO matrix) are associated to occupied intragap states at the valence band top, and up to 0.9 eV above the valence band edge. Residual S may be responsible for compensating effects of (unintentional) *n*-character of the host, or even to possible *p*-type doping.

## Results and Discussion

### Morphological, Structural and optical properties: effect of S inclusions

**Figure 1:**
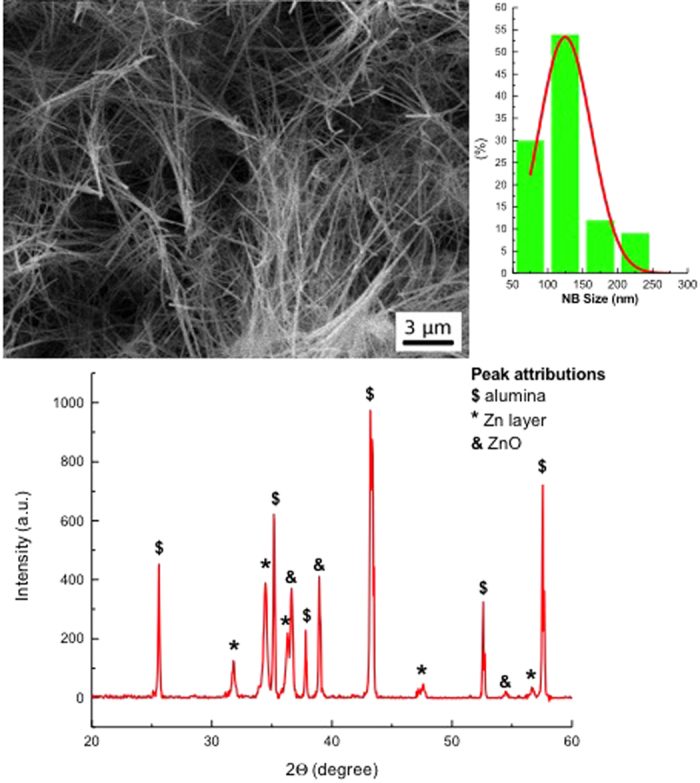
(**a**) SEM image of the NBs, (**b**) the size statistics is reported, showing that the average diameter is between 100 and 150 nm. (**c**) XRD pattern of the ZnO nanobelts grown on alumina substrates covered with a zinc layer.

The morphological analysis, performed by secondary electron microscopy (Fig. 1a), reveals that the process results in an homogeneous growth of ZnO nanobelts on the alumina substrate covered with a zinc layer. Nanobelts are long tens of micron. The lateral size distribution demonstrates that the NBs width ranges from 50 nm up to 250 nm with a tail of few nanobelts with a lateral size larger than 500 nm. The size distribution is peaked at 125 nm, shown in Fig. 1b. Figure 1c shows the X-Ray Diffraction pattern (XRD) of the ZnO nanobelts, where it is possible to identify the peaks related to the zinc covered alumina substrates and the peakes related to the ZnO nanostructures.

**Figure 2:**
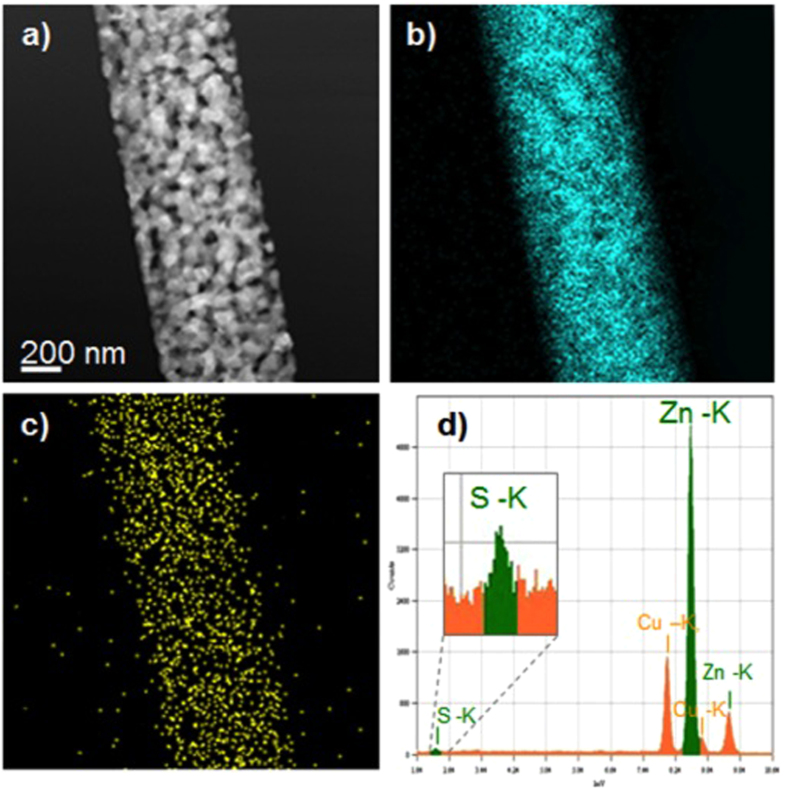
(**a**) STEM image of a nanobelt; (**b**) Zn map; (**c**) S map; (**d**) EDS spectrum from the same area. The Cu-K peaks are due to the Copper grid used for TEM analysis.

Figure 2 shows the STEM analysis of a single nanobelt. The STEM image reveals the mesoporous morphology of the single nanobelt. The nanobelt width is about 600 nm with an average grain size of about 80 nm. The *compositional* information is obtained using *EDS mapping and spectroscopy.* Figure 2b,c presents the zinc and the sulfur maps, respectively. As highlighted in Fig. 2c, sulfur is detected with a homogeneous distribution over the entire nanobelt. Quantitative analysis gives a concentration of S equal to 0.7% obtained by the EDS spectrum, reported in Fig. 2d. The copper signal is due to the TEM grid used in the experiments.

**Figure 3:**
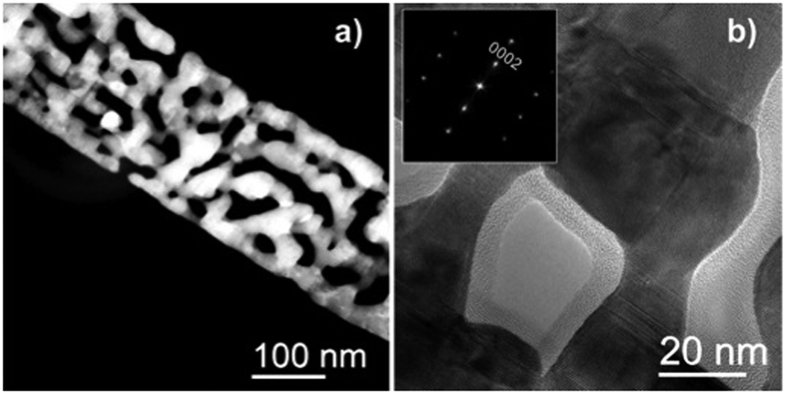
(**a**) High-Angle Annular Dark Field (HAADF) and (**b**) High Resolution TEM (HRTEM) images of a NB. The Fast Fourier Transform of the HRTEM image (inset) shows that the mesoporous ZnO nanobelts are single crystals oriented along the [0001] direction.

The evaluation of the ZnO NBs crystallinity is carried out by high resolution TEM as reported in Fig. 3. Figure 3a, a Dark Field TEM image, shows the presence of voids along the nanobelts, demonstrating high surface-to-volume ratio of the single nanobelt. In addition Fig. 3b demonstrates that the nanobelts are single crystals, affected by line defects, mainly stacking faults. HRTEM analysis of the nanobelts reveals that the ZnO wurtzite lattice is not perturbed by the high content of sulfur, as shown by the Fast Fourier Transform (FFT) shown in the inset of Fig. 3b. The HRTEM lattice resolved image (not shown here) demonstrates the lattice spacing of 0.52 nm in accordance with the *c* lattice constant of bulk ZnO (0.52 nm). This supports the hypothesis of the substitutional position of sulfur atoms in the ZnO lattice.

**Figure 4:**
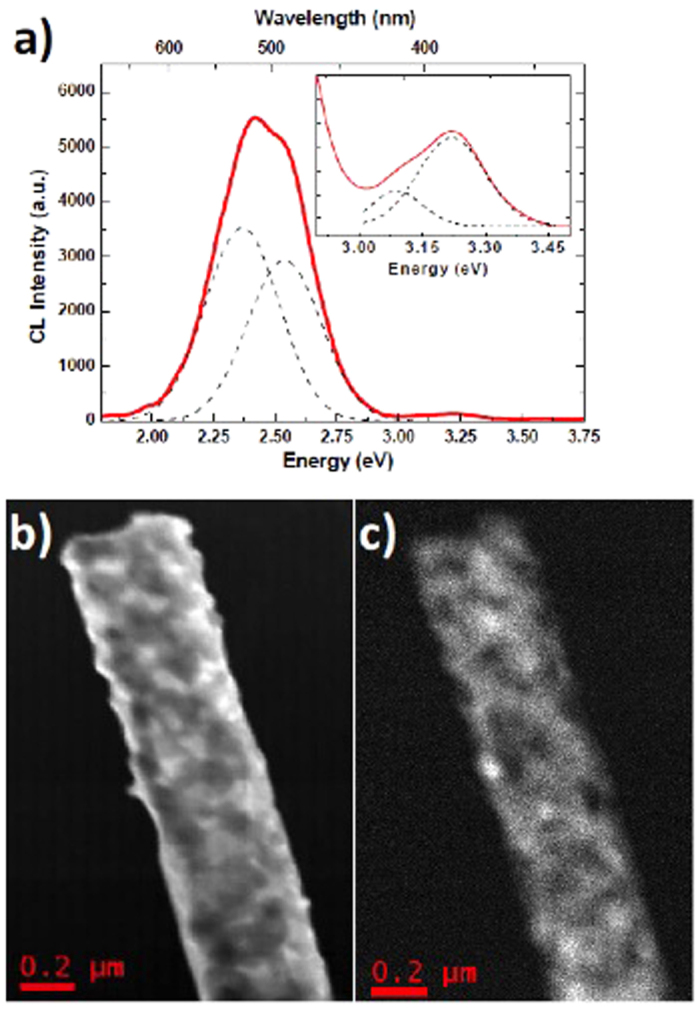
(**a**) STEM-CL spectra of a single ZnO NB. In the inset an enlargement of the UV range is shown. (**b**) Dark Field STEM image of the NB in analysis; (**c**) Panchromatic STEM-CL map.

The study of the light-emission properties of single S-doped ZnO nanostructures is carried out by STEM-CL at room temperature (Fig. 4). The RT STEM-CL spectrum of single NB presents two bands centered at 3.2 eV and 2.4 eV. The higher energy emission (Inset of Fig. 4a) is composed of two different components peaked at 3.09 eV and 3.22 eV, respectively, in good agreement with what reported for the unintentionally doped bulk ZnO near-band-edge (NBE) emission at room temperature^15^. At variance with previous experimental results^13^,^14^, no further S-related shallow level can be resolved in the optical spectra, therefore we can infer that sulfur dosages up to ~1% concentration do not induce any ZnO band-gap narrowing, in agreement with previous works^10^. The broad visible emission is composed of two different peaks, set at 2.36 eV and 2.5 eV. The 2.5 eV emission is assigned to the green band and related to intrinsic surface defects in ZnO^15^. The 2.36 eV band is instead assigned to the sulfur inclusions, in agreement with different light emission studies showing a radiative center at about 2.4 eV^16^ and a luminescence blue-shift with increasing the sulfur content^12^.

Figure 4b shows the dark-field STEM (DF-STEM) of the single NB where the CL spectrum is obtained. The panchromatic STEM-CL map, reported in Fig. 4c, reveals that the light emission intensity shows inhomogeneities that can be related to the NB morphology and in particular to the local thickness of ZnO. A comparison of the DF-STEM contrast and the panchromatic STEM-CL map intensity, indeed, clarifies that the light emission is more intense where the nanobelt is thicker (consider that this dark field STEM is not an High Angle Anular Dark Field TEM image, so the contrast results darker in the thicker areas.). This effect is due to the electron excitation that is more efficient for the thicker areas of the samples^17^, i.e. where the nanobelt is thicker. By assuming a uniform density of defects, the amount of defects should be higher, supporting a more intense light emission yield.

The experimental results demonstrate that a 0.7% concentration of residual sulfur does not modify the structural and optical properties of mesoporous ZnO nanobelts: (i) the ZnO *c* lattice constant and (ii) the ZnO NBE emission results in agreement with what previously reported for undoped ZnO one-dimensional nanostructures^15^,^18^.

**Figure 5:**
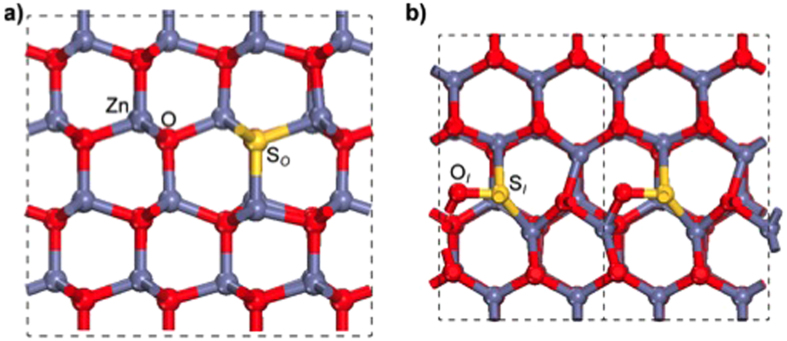
The relaxed atomic structures of S-doped ZnO in the simulation supercell in (**a**) substitutional (SO) and (**b**) interstitial (SI) geometries. Red (grey) balls indicate O (Zn) atoms, while the yellow balls represent S atoms. Visible local bond distortions accompany S inclusion in the ZnO matrix, because of its larger atomic radius with respect to O. Two different orientations (along -a- and perpendicular -b- the polar axis) are represented to highlight different features; unitary cell in panel b is replicated for clarity.

**Figure 6:**
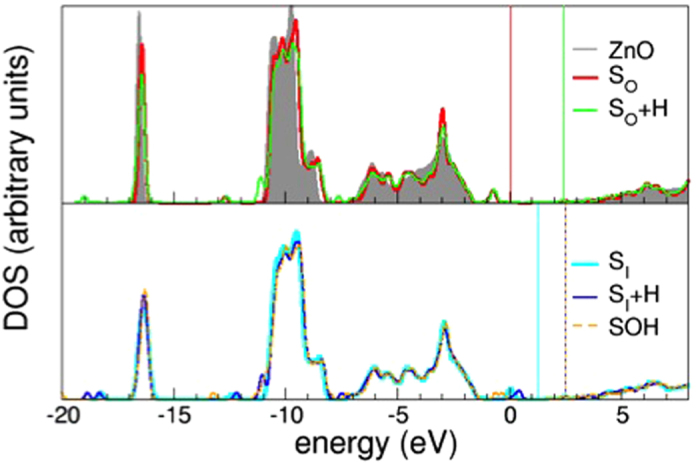
Total DOS of ZnO (grey line, shaded area), compared to the doped S:ZnO bulk, (SO/SI in red/cyan line). The effect of interstitial H inclusions to simulate *n*-doping of the host is also reported (green line for neutral SO+H, blue for neutral SI+H, and orange for the SOH complex). The zero of the energy scale is the Fermi level of the clean bulk ZnO, while vertical colored lines mark the Fermi energy of the defected systems, respectively. The sample character is not affected by neutral S defects.

We can interpret the aforeshown experimental results on the basis of the theoretical predictions of Density Functional Theory. To get a one-to-one understanding of the effect of sulfur, we adopted a simplified model, representative of the experimental system (see Fig. 5): bulk calculations of S inclusion in substitutional O sites have been performed at similar amounts of sulfur as evaluated by EDX studies (~0.8–1.6%). The overall experimental sample dimensions, that present ZnO contiguous portions of several hundreds of nm^3^, can be properly reproduced by a bulk simulation; the presence of a surface or interface does not induce remarkable effects: a discussion of the ZnO/ZnS interface is reported in Supplementary Information (SI) for completeness. At these defect contents, upon atomic relaxation (see Fig. 5a), we observe a small local rearrangement of atomic coordinates around the dopant site, induced by the large atomic radius of S compared to O. The bond distortion close to the defect site is accompanied by charge rearrangements that are responsible for an occupied extra filled peak mid-gap in the DOS, ~0.8 eV above the host valence band top, and a further low lying filled state in the pristine ZnO ionicity gap (~−12.8 eV) as depicted in Fig. 6 (red line). An analysis of the Projected Density of States (PDOS - not shown) reveals that the peak fully derives from S which is less electronegative than O, thus less capable to capture Zn-charge in the formation of the bond. This is also confirmed by a slightly more atomic-like Löwdin charge for S (7.12) than the O atoms (7.32) in the host. Thus, albeit isoelectronic to oxygen, the presence of sulfur atoms induces the formation of less-bonded states (i.e. lower binding energy) than the corresponding oxygen ones, which constitute the valence band maximum (VBM) of clean ZnO. This results in the appearance of a fully occupied S-derived state above VBM, which position is in very good agreement with the experimental findings. Furthermore, the energy level of this feature is consistent with the staggered, type II alignment of band at the ZnO/ZnS interface (see SI)^19^.

Because of the specific experimental conditions, where ZnO NBs have been obtained through replacement of S atoms by O atoms during thermal treatments in air, susbtitutional neutral S_O_ should be the most probable S-related defect in the ZnO matrix, in agreement with both TEM and calculated defect formation energies (The defect formation energy for the neutral interstitial sulfur atom SI is 0.7 eV higher that the substitutional defect SO). For completeness, we have anyhow considered also interstitial S defects and the effect of unintentional *n* doping. Neutral interstitial sulfur atoms (S_I_) produce a much larger distortion in the ZnO lattice (Fig. 4b): S_I_ almost substitutes an oxygen atom (O_I_), which is displaced in an interstitial site, confirming the attitude of S to assume O-substitutional configurations. The simultaneous interaction with nearest-neighbor Zn and O_I_ atoms promotes a shift of the S-related extra-peak at higher energies in the band gap (Fig. 5, bottom panel). Both in the substitutional and interstitial configurations these are filled peaks at ~0.8–1 eV above the ZnO VBM: this effect is not consistent with the interpretation of a possible role in *n*-doping for S^13^.

### Codoping in the presence of H

In the experiment, the samples are not grown in presence of explicit (i.e. intentional) *n*-dopants. ZnO however usually presents *n*-character^20^. Interstitial H is recognized as the most probable origin of unintentional *n-*doping in bulk ZnO^15^,^20^,^21^,^22^,^23^. Furthermore, H is largely compatible with the present experiment, since it is extremely probable that ZnO samples heated in an open furnace can incorporate H. Furthermore, residual hydrogen may also be present in the samples for incomplete dissociation in the reaction *ZnS(en)*_*0.5*_ → *ZnS* → *ZnO.* In order to provide a more strict and predictive comparison with the experimental situation, we have also considered the combined effect of S and H doping. We thus performed selected calculations for a ZnO supercell containing interstitial H and an equivalent dosage of S inclusions in different charge states and structural configurations.

Our results show that the sample character is not affected by neutral S defects (see Fig. 6), irrespective of the relative position in the host matrix, either substitutional or interstitial. The presence of H has no effect on the stability of the defects, thus on their probability, in terms of formation energy ordering. The neutral susbtitutional S_O_ remains the most stable defect, while the electronic properties of the two defects, interstitial H and S_O_/S_I_, sum-up without relevant changes. If S_I_ and H are sufficiently close in the host, they may give rise to a SH complex that eventually binds to an O atom (SOH complex). Although highly stable (0.5 eV lower in energy than the isolated defect), this structure has no remarkable effect on the electronic properties of the system (orange dashed line in Fig. 6).

**Figure 7:**
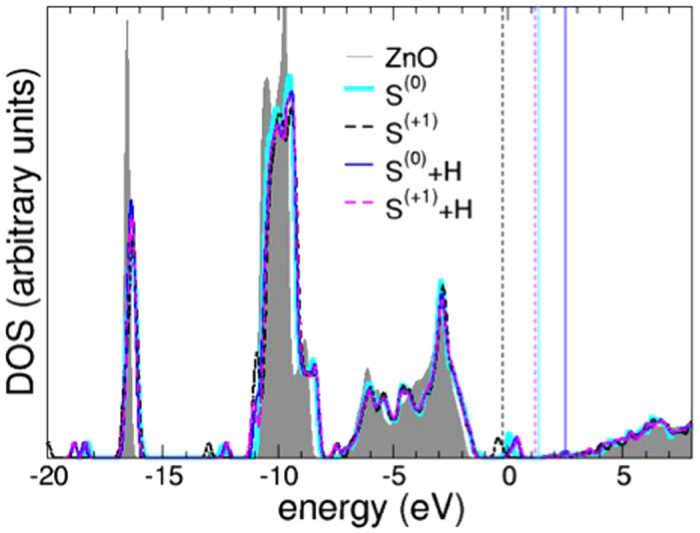
Total DOS of ZnO (grey line, shaded area), compared to ZnO bulk, in the presence of interstitial H to simulate *n*-doping of the host (blue line for neutral S(0)+H, magenta line for S(+1)+H). The zero of the energy scale is the Fermi level of the clean bulk ZnO, while vertical colored lines mark the Fermi energy of the defected systems, respectively. Charge states are explicitly reported in parenthesis for clarity.

On the other hand, the presence of S in different defect ionization states can severely alter the situation. While the occurrence of negative charged states (S^(−1)^, S^(−2)^) does not lead to any compensating effect of *n*-type character, if sulfur is found in a positive charged state (the most probable is S^(+2)^) in the otherwise neutral ZnO matrix, the feature associated with the defect mid-gap in the DOS (S^(+1)^) or right above VBM (S^(+2)^) is a half-filled peak, thus the system is a *p*-type conductor (see Fig. 7). In case of coexistence S^(+1)^, S^(+2)^ and unintentional donors (e.g. H), the extra-charge donated is captured by S^+^ ions, and charge neutrality of the whole sample can be restored, depending on the oxidation states and number of donors, driving the situation towards a neutral-like system: the mechanism is illustrated in Fig. 7 for S^(+1)^, as a representative case. S can act thus as compensating agent, if it occurs in positively charged states. This result corroborates the present experimental study, and the experimental and theoretical analysis in ref. 10, where a role in *p*-codoping with N was linked to the presence of S.

In conclusion, residual sulfur inclusions close to 1% concentration with homogeneous distribution have been found to be stable in the ZnO porous matrix. S-associated defects in ZnO induce an optically active intra-gap state at about 0.9 eV above the valence band maximum, with an optical emission at 2.36 eV as obtained by Density Functional Theory and cathodoluminescence spectroscopy, respectively. DFT confirms that the experimentally found energy level can be preferentially associated to substitutional sulfur atoms in oxygen position. First-principles simulations predict a role as compensating agent and a possible *p*-type doping for S inclusions in ZnO.

## Methods

### Experimental

The sample syntheses^24^ were performed by adding S powder (0.25 mmol), KBH4 (0.75 mmol), and ethylenediamine (*en* - 20 mL) in a commercial Teflon-lined autoclave with a capacity of 45 mL. The mixture was stirred vigorously on a magnetic stir plate for 5 min on an alumina substrate where Zn metal patterns were previously defined by standard photolithography and lift off procedures so that the Zn area was kept to 1.5 cm^2^ 25. The metallized alumina substrate was then inserted into the liner, the autoclave was sealed, maintained at 200 °C in an oven for 24 h to grow ZnS(*en*)_0.5_ hybrid nanobelts, and finally cooled naturally to room temperature. The substrate with synthesis products was removed from the solution, washed by rinsing in ethanol and de-ionized water and dried overnight in an oven at 65 °C. Samples were then annealed in air first at 400 °C for 50 h and then at 600 °C for 2 h. This procedure allowed S and *en* evaporation and substitution with O, thus giving rise to porous ZnO nanobelts.

Scanning transmission electron microscopy energy-dispersive X-ray spectroscopy (STEM-EDS) is carried out at 200 kV in a field-emission JEOL 2200FS microscope equipped with Omega filter.

STEM-CL is carried out in a JEOL 2011 equipped with a Gatan MONOCL3 system for serial and parallel acquisition^26^. STEM-CL spectroscopy and mapping are carried out at RT with an accelerating voltage of 80 keV and a beam current of 2 nA.

### Simulations

Electronic structure analysis is based on Density Functional Theory (DFT) simulations at the PBE-GGA^27^ level of approximation, as coded in the Quantum ESPRESSO package^28^. Ultrasoft pseudopotentials^29^ are used for all of the atomic species; single particle wavefunctions (charge) are expanded in plane waves up to an energy cutoff of 28 (280) Ry. The *3d* electrons of Zn have been explicitly included in the valence shell. The DFT bandstructure was corrected by including an ad hoc Hubbard potential on both *3d* orbitals of zinc (U_Zn_ = 12.0 eV) and *2p* orbitals of oxygen (U_O_ = 6.5 eV), along the lines previously described in ref. 30. Numerical details and accuracy tests on the effects of the DFT+U approach on the structural and electronic properties of ZnO are reported elsewhere^30^. This is an efficient and computationally inexpensive way to correct for the severe underestimation of the bandgap and the wrong energy position of the *d*-bands of the Zn atoms^31^,^32^,^33^. We considered different configurations for S in the ZnO bulk matrix: first of all, susbtitutional S doping in O sites (S_O_) or interstitial S (S_I_) with doping level of ~0.8–1.6% (1/128–1/64 atoms), very close to the experimental detected ones, were studied. Selected calculations were also performed for charged states of interstitial defects, with/without the presence of interstitial H to account for unintentional *n*-doping of the ZnO host. Bulk calculations are performed in periodically repeated orthorhombic supercells, including up to 128 atoms, as shown in Fig. 1. A (4 × 4 × 4) k-point grid is used to sample the complete Brillouin zone. All structures were relaxed until forces on all atoms were lower than 0.03 eV/Å.

A different set of calculations was performed via ab initio molecular dynamics to evaluate the possible intermixing effects at finite temperatures, close to the experimental conditions. An account of interface simulations is presented in SI.

## Additional Information

**How to cite this article**: Fabbri, F. *et al*. S-induced modifications of the optoelectronic properties of ZnO mesoporous nanobelts. *Sci. Rep.* **6**, 27948; doi: 10.1038/srep27948 (2016).

## Supplementary Material

Supplementary Information
