# Supplementary material for: S-induced modifications of the optoelectronic properties of ZnO mesoporous nanobelts
Source: Sci Rep. 2016 Jun 15;6:27948. doi: 10.1038/srep27948 (PMC4995295; doi:10.1038/srep27948)
Supplement: Supplementary Information [file srep27948-s1.pdf]

S-induced modifications of the optoelectronic properties of ZnO mesoporous nanobelts

Supplementary Information

Filippo Fabbri*[1,2], Lucia Nasi[1], Paolo Fedeli[1], Patrizia Ferro[1], Giancarlo Salviati[1], Roberto Mosca[1], Arrigo Calzolari[3], Alessandra Catellani*[1,3]

1 CNR-IMEM, Parco Area delle Scienze 37a, I-43124 Parma, Italy

2 KET Lab, c/o Italian Space Agency via del Politecnico, 00133 Roma, Italy

3 CNR-NANO, Istituto Nanoscienze, Centro S3, 41125 Modena, Italy

Ab initio simulations of a ZnO/ZnS (1010) interface

With the same level of accuracy of the simulations presented in the main text, we studied a ZnO/ZnS (1010) interface. The simulation supercell is in this case composed of 4 ZnO bilayers with (2x1) two-dimensional periodicity terminated by a ZnS bilayer at both sides: the slab replicas are separated by an equivalent vacuum region (~10 Å). Calculations were performed on a regular (4x4x1) *k*-point mesh of the supercell Brillouin Zone, with 28Ry cutoff on plane wave expansions. All atoms were relaxed until forces were lower than 0.03 eV/A.

In the left panel of Fig. S1 the electronic properties of bulk (top) and interface (bottom) calculations are presented for comparison. In both cases, the energy reference has been put to the Fermi level of the specific system. The apparent valence band offset (bottom panel) between ZnO and ZnS is consistent with a type II alignment as predicted by experiment and different calculations.

For the free interface on the same supercell we further performed ab initio molecular dynamic

simulations at finite temperature, T~400K, close to the experimental preparation conditions; here, calculations were performed at the G point of the supercell, with 28Ry cutoff on plane wave expansions. All atoms were allowed to move under the effect of Hellmann-Feynman forces; the full cycle simulation run was ~1.3ps. A snapshot of the atomic distribution in the supercell during the simulation run is shown in the rightmost panel of Fig. S1. At these temperatures, the ZnS bilayer is largely distorted, with tendency to evaporating/detaching from the substrate, however the inner S atoms still form loose bonds with the ZnO substrate: these bonds are responsible for features at the interface DOS valence band edge, also present for the abrupt interface (Fig. S1).

The presence of these structures above the valence band maximum is consistent with the position of the S-derived peak for an S impurity in the ZnO bulk matrix, as shown in the main text and reported here (Fig. S1) as topmost left panel.

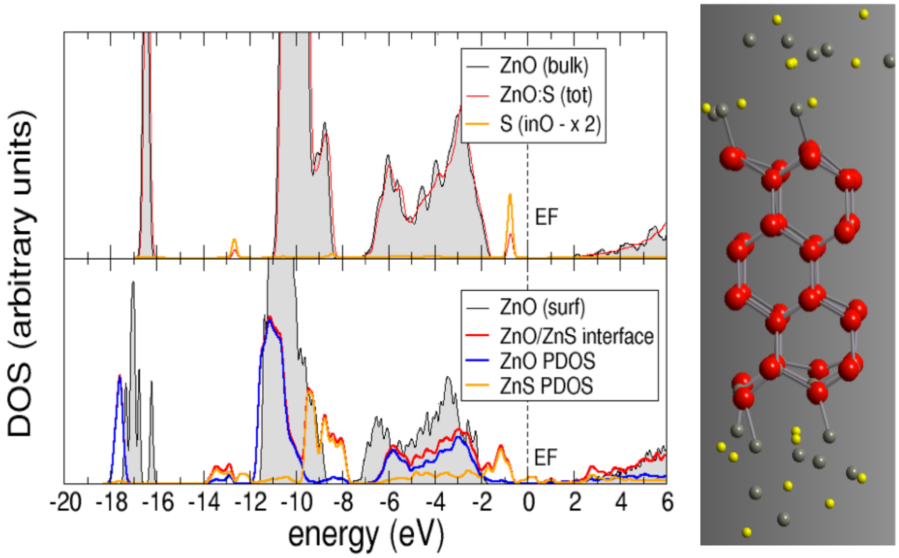

Fig. S1: Electronic structure (Density of states) of a ZnS/ZnO interface as obtained by ab initio calculations. A snapshot of the free interface evolution during an ab initio molecular

dynamics simulation at 400K is shown on the right: here, red (grey) balls indicate O (Zn) atoms, while the yellow balls stem for S atoms.

References

1. C. Persson, et al, *Phys. Rev. Lett.* **97**,146403 (2006).

2. Wei, S.-H.; Zunger, A. *Appl. Phys. Lett. 72*, 2011 (1998).
